# Unveiling the Impacts of Sodium Hypochlorite on the Characteristics and Fouling Behaviors of Different Commercial Polyvinylidene Fluoride Hollow Fiber Membranes

**DOI:** 10.3390/membranes12100965

**Published:** 2022-10-01

**Authors:** Muqiao Han, Qi Han, Shanwei Wu, Hu Xiao, Lei Zhang, Yibo Lin, Fangang Meng, Shanshan Zhao

**Affiliations:** 1 School of Environmental Science and Engineering, Sun Yat-sen University, Guangzhou 510006, China; 2 Guangdong Provincial Key Laboratory of Environmental Pollution Control and Remediation Technology, Guangzhou 510006, China; 3 Guangzhou Jinrongtao Environmental Protection Technology Co., Ltd., Guangzhou 511400, China

**Keywords:** NaOCl cleaning, PVDF hollow fiber membrane, physicochemical properties, membrane fouling behavior

## Abstract

Sodium hypochlorite (NaOCl) is a commonly used cleaning agent for recovering membrane performance in membrane technologies. A thorough understanding of the impacts of NaOCl exposure on membrane properties and fouling behavior is important for optimizing chemical cleaning process and extending membrane lifespan. In this study, three commercial polyvinylidene fluoride (PVDF) hollow fiber ultrafiltration membranes (SMM-1010, MEMCOR^®^ CS II and ZeeWeed 500) were used to systematically explore the effects of NaOCl dose and solution pH (8 and 10) on membrane properties. The results showed that membrane pores increased with exposure time prolonging, and more pores were observed at pH 8 aging condition. The amide group in the Fourier transformation infrared spectra was disappeared, while the carboxylic acid and succinimide groups were formed at pH 10 and pH 8 conditions, respectively. The hydrophilicity and pure water permeability (PWP) of SMM-1010 and MEMCOR^®^ CS II membranes had insignificant changes during NaOCl aging process, whereas the hydrophilicity of ZeeWeed 500 membrane slightly decreased and its PWP increased by 1.4-fold. The antifouling properties of NaOCl-aged SMM-1010 and MEMCOR^®^ CS II membranes were slightly improved, whereas the NaOCl-aged ZeeWeed 500 membrane showed severer flux decline with humic acid filtration. Our findings could provide guidance for practical chemical cleaning process optimization.

## 1. Introduction

Over the past two decades, membrane bioreactor (MBR) technology has aroused great attention and has been increasingly used for water and wastewater treatment worldwide because of its merits of high effluent quality, low sludge production, small footprint, and easy operation [1,2]. For example, the total capacity of large-scale MBR in China increased from 1 million m^3^/d in 2010 to 12 million m^3^/d in 2018, and the number of large-scale MBR treatment plants has exceeded 300 [3]. However, membrane fouling is still an inevitable problem in MBR, posing a great challenge for the wide application of this technology. The accumulation of organic, inorganic, or microbial foulants on membrane surface or within membrane pores not only reduce the effluent quality and water productivity, but also increase maintenance cost and deteriorate membrane lifespan [1,4,5]. Therefore, in order to recover membrane performance, chemical cleaning is routinely operated to remove membrane foulants.

Among all the chemical cleaning agents, sodium hypochlorite (NaOCl) is the most commonly used one in MBR due to its advantages of low price and high efficiency for removing organic/biological fouling [6,7]. In addition, NaOCl is also extensively employed for the cleaning of microfiltration and ultrafiltration membranes used in water and wastewater treatment [8,9]. Nevertheless, NaOCl cleaning is known to possibly deteriorate the intrinsic properties of the membrane, which might reduce the long-term operation performance of MBR and shorten membrane lifespan. During the past ten years, many efforts have been paid to study the impacts of NaOCl cleaning on the physicochemical properties and separation performance of membranes [10,11,12,13,14,15,16,17,18]. For instance, the hydrophilicity, pore size, and surface negative charge intensity of the polysulfone (PSF) membrane were reported to be increased after NaOCl aging, which led to an increase in the rejection of bovine serum albumin (BSA) but decrease in the rejection of humic acid (HA) [18]. The dynamic aging process of the polyethersulfone/polyvinylpyrrolidone (PES/PVP) membrane during NaOCl exposure has been investigated using two-dimensional Fourier transformation infrared (FTIR) correlation spectroscopy. The oxidation and dissolution of hydrophilic additive PVP followed by chain scission of C-S bond was identified as the main aging mechanism of the PES membrane, which resulted in enlargement of pore size and increase in water permeability [19].

Polyvinylidene fluoride (PVDF) membranes, with their merits of outstanding chemical resistant, good thermal and mechanical stability, are widely used in different fields, such as water and wastewater treatment, gas separation, lithium battery, and bioethanol recovery [20]. More importantly, PVDF membrane is the most commonly used polymeric membrane in MBR. Therefore, a thorough understanding of the impacts of NaOCl cleaning on the characteristics and performances of PVDF membranes is essential. Several researches have reported that the roughness and pore size of PVDF membrane were increased after exposure to NaOCl, however, its surface hydrophilicity has been reported to be either increased or decreased or even first increased and then decreased with the NaOCl exposure time prolonging [11,12,15,17,18]. It seems that the impacts of NaOCl on the membrane hydrophilicity needs further verification. In addition, most of these studies mainly focused on changes in intrinsic characteristics of the aged membranes, however, few studies correlated these physicochemical property changes to their fouling behaviors [12,17,18]. Actually, the changes in pore size, surface functionality and surface hydrophilicity may play significant roles in the fouling behavior or antifouling performance of the NaOCl-aged membranes, and they should be focused on [21,22,23]. Zhang et al. reported that the flux of PVDF membrane declined slower with HA filtration while the flux decreased faster with BSA filtration after exposure to 400 mg/L NaOCl solution for 100 h [18]. However, the changes in fouling behaviors of NaOCl-aged PVDF membrane with various exposure time should be systematically investigated. Moreover, it has been demonstrated that the oxidation ability of NaOCl depends on solution pH [19], which would influence the interaction between NaOCl and PVDF membrane. Therefore, the impacts of NaOCl solution pH on the membrane properties and fouling behaviors should also be investigated. 

In this study, three widely used commercial PVDF hollow fiber membranes (SMM-1010, MEMCOR^®^ CS II, and ZeeWeed 500) were employed to explore the impacts of NaOCl ageing on membrane properties and fouling behaviors. Specifically, the exposure dose and solution pH were considered. The changes in membrane morphologies, surface functional groups, water contact angles, and mechanical properties with the NaOCl exposure time and pH were first systematically examined. Then, the water permeability, HA rejection, and fouling behaviors of these NaOCl-aged membranes were tested and compared. The changes in membrane physicochemical properties and fouling behaviors would be correlated and discussed to understand the NaOCl ageing process. Our work could provide useful guidance for practical chemical cleaning.

## 2. Materials and Methods

### 2.1. Membranes and Chemicals 

Three commercial PVDF hollow fiber ultrafiltration (UF) membranes commonly used in MBRs were purchased from different manufacturers (more details are shown in Table 1), which were denoted as A, B, and C, respectively. Sodium hypochloride (NaOCl) (AR-grade, available chlorine 6–14%) was purchased from Aladdin (Shanghai, China). Humic Acid (HA) (fluvic acid ≥ 90%) was provided by Macklin Biochemical Co., Ltd (Shanghai, China). 1 mol L^−1^ HCl and NaOH (AR-grade, Guangzhou chemical reagent factory, Guangzhou, China) solutions were used to adjust the pH of NaOCl aqueous solution. The ultrapure water provided by a Milli-Q^®^ integral water purification system (spring-R2+Omni(genet), Xiamen, China) was used to prepare all aqueous solutions.

### 2.2. NaOCl Exposure Experiments

Before the NaOCl exposure experiments, all of the hollow fiber membranes were rinsed to remove any pore preservation agent and impurities. Specifically, A and B membranes were immersed in the ultrapure water for 72 h with water being refreshed every 24 h. C membranes were first immersed in 25 vol.% ethanol for 0.5 h followed by ultrapure water rinsing for three times (30 min for each time), and then the hollow fiber membranes were immersed in the ultrapure water for 72 h with the water being replaced for several times.

After removing impurities, the A, B and C membranes were separately soaked in 3 L NaOCl solutions (2000 ppm total free chlorine, pH = 10, 25 ± 2 °C) in three sealed containers in the dark for 0~500 h. Normally, the NaOCl concentration for in situ maintenance cleaning is 300~2000 mg/L and the maintenance duration is 0.5~4.5 h [24]. Therefore, relative higher NaOCl concentration 2000 mg/L was selected to accelerate the membrane aging process. The exposure dose was determined as NaOCl concentration multiplied by exposure time. The maximum cumulative exposure dose of 1,000,000 ppm·h corresponds to the maximum dose recommended by the membrane manufactures [9]. In addition, the A membranes were also immersed in the NaOCl solution with the same concentration but different pH (pH 8.0) in order to investigate the effects of NaOCl solution pH on the membrane characteristics. Since the NaOCl solutions with a pH below 7 have been reported to have a strong oxidation capability to membranes, only weak alkaline conditions (pH 8 and 10) were considered in this study [19,25]. The NaOCl solutions were refreshed every 48 h to keep a constant free chlorine concentration and solution pH. The free chlorine concentration was determined by the *N,N*-diethyl-*p*-phenylenediamine (DPD) method [26]. All sampled membranes were thoroughly rinsed with ultrapure water prior to further characterization.

### 2.3. Membrane Characterization Techniques

The surface morphologies of the pristine and NaOCl-exposed membranes were observed via a high-resolution cold field emission scanning electron microscopy (CFESEM) (Regulus 8230, HITACHI, Tokyo, Japan) while their cross-section morphologies were examined through a field emission scanning electron microscopy (FESEM) (Quanta 400 FEG, FEI, Hillsboro, OR, USA). Changes in the surface functional groups of the PVDF hollow fiber membranes after NaOCl exposure were characterized by a Fourier transform infrared spectrometer with an attenuated total reflectance accessory (ATR-FTIR) (Vertex70Hyperion3000, Bruker, Karlsruhe, Germany). Each spectrum was recorded in a scan rang of 400–4000 cm^−1^ with a resolution of 4 cm^−1^. The dynamic water contact angles (WCAs) of the NaOCl-exposed membranes were measured using a force tensiometer (K100, Krűss, Hamburg, Germany) based on Wilhelmy method [27]. A single hollow fiber was attached to the sample holder and kept perpendicular to the liquid surface. Then, the changes in forces during the fiber immersing into and withdrawing from the liquid were measured by the microbalance. The advancing and receding velocities were set as 5 mm/min and the immersion depth was set as 5 mm. Before the dynamic water contact angles determination, the wetted length (*L*) of the hollow fiber was measured using n-hexane. After that, n-hexane was changed to pure water, and the dynamic water contact angle was detected according to the equation $cos\theta =\frac{F}{\gamma L}$, where *θ* is the dynamic water contact angle, *F* is the force detected by the microbalance, *γ* is the liquid surface tension, and *L* is the wetting length of the hollow fiber. At least three segments of hollow fibers from different locations were tested for each membrane sample to obtain the averaged value. All the membrane samples were freeze-dried under vacuum by a Freezer Dryer (10A, Biosafer, Nanjing, China) prior to these characterizations. 

The mechanical properties of A and B membranes were tested by an Instron Universal Testing System (5943R7985, Instron, Boston, MA, USA) with an initial length of 55 mm at an elongation rate of 250 mm/min. At least six fibers were tested for each NaOCl exposure condition, and the averaged values were provided. Since C membranes have reinforced the support layer, their mechanical properties were assessed by measuring the collapse pressure of the membrane modules. Each membrane module consisted of 5 fibers with an effective length of 24 cm. The collapse pressure was defined as the hydraulic pressure applied on the shell side of the modules until a sudden change in the permeate flux. The hydraulic pressures at the interval of 1–6 bar was applied with 0.5 bar increase every 10 min. 

### 2.4. Membrane Separation Performance and Fouling Behavior Evaluation

The effects of NaOCl exposure on membrane separation performance and fouling behavior were evaluated in a bench-scale crossflow filtration system (Figure S1). All of the membrane modules were tested in an outside-in mode because the membrane skin layers were on the shell side of the hollow fibers. The membrane modules were first pre-compacted using ultrapure water under a pressure of 1 bar at 25 ± 1 °C for 1 h. Then, the pure water permeability (PWP, *J*_0_) of the membranes was measured under the pressure of 0.5 bar at 25 ± 1 °C, which can be calculated using Equation (1).

(1) $$J_{0}=\frac{V}{A\times \Delta t\times \Delta P}$$

where, *J*_0_ (L/(m^2^·h·bar), LMH/bar) is the PWP, *V* (L) is the volume of collected permeate solution, *A* (m^2^) is the effective membrane area (the effective membrane areas for A, B, and C modules are 0.0060, 0.0053, and 0.0048 m^2^, respectively), and Δ*t* (h) and Δ*P* (bar) are the sample collection time and transmembrane pressure, respectively. 

The fouling behavior of NaOCl-exposed membranes was evaluated using 2 L HA solution with a concentration of 50 mg/L-dissolved organic carbon (DOC) as feed. The feed temperature was maintained at 25 ± 1 °C using a circulator bath. The filtration was conducted under a pressure of 0.5 bar for 1 h at a crossflow velocity of 0.25 m/s. The permeate flux (*J_w_*) was measured simultaneously by recording the mass change in the permeate solution via a balance (LQ-A20002, Lucky, Yixing, China). The change in normalized flux (*J_wt_/J*_0_) with filtration time was used to express the membrane fouling behavior. Where *J_wt_* and *J*_0_ were the permeate flux of HA solution during filtration and the initial pure water flux, respectively. At the end of the filtration, the concentrated and permeated solutions were collected to measure HA concentration. The HA rejection can be calculated using Equation (2).

(2) $$R=(1-\frac{C_{P}}{C_{F}})\times 100\%$$

where *R* (%) is the HA rejection, *C_P_* (mg/L) and *C_F_* (mg/L) are the DOC concentrations of the concentrate and permeate solutions, respectively. The DOC concentration of HA was measured by a total organic carbon analyzer (TOC-L) (CPH, SHIMADZU, Japan).

After the fouling experiment, the fouled membranes were rinsed with ultrapure water at a crossflow velocity of 0.25 m/s for 15 min (the water was refreshed every 5 min) without pressure. Finally, the cleaned membranes were pre-compacted using ultrapure water under pressure of 1 bar for 0.5 h before PWP measurement under pressure of 0.5 bar. The PWP of the cleaned membrane was denoted as *J*_2_. Then, the flux recovery ratio (FRR) was determined using (*J*_2_/*J*_0_) × 100%.

## 3. Results and Discussion

### 3.1. Effects of NaOCl Exposure on Membrane Physicochemical Characteristics

The physicochemical characteristics of membranes are closely related to their separation performance and fouling behavior. Thus, the effects of NaOCl exposure on the physicochemical characteristics of the PVDF membranes, such as surface and cross-section morphologies, surface functional groups, hydrophilicity, and mechanical properties, were first systematically investigated and discussed. 

#### 3.1.1. Surface and Cross-Section Morphologies of the NaOCl-Aged Membranes 

**Figure 1:**
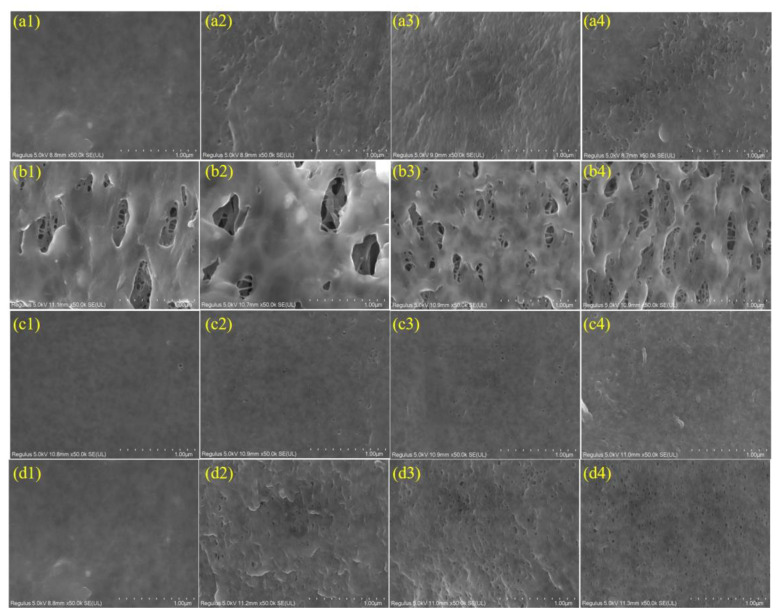
FESEM images of the outer surface morphologies of the pristine and NaOCl-exposed membranes. (**a1**–**a4**) A membrane, NaOCl Ph = 10; (**b1**–**b4**) B membrane, NaOCl pH = 10; (**c1**–**c4**) C membrane, NaOCl pH = 10; and (**d1**–**d4**) A membrane, NaOCl pH = 8; 1, 2, 3, and 4 represent exposure time 0, 48, 192, and 500 h, respectively.

The surface morphologies of the pristine PVDF and NaOCl-aged membranes were shown in Figure 1. Dense skin layers were observed on both A and C pristine membrane surfaces while porous structure was observed on B membrane surface. The surface morphologies changed significantly after NaOCl aging experiments. A wrinkle structure and abundant pores appeared on the A membrane surface after exposure to NaOCl. The number of pores increased with the exposure time increasing. In addition, compared with NaOCl pH 10 condition, more pores were observed on the A membrane surface after exposure to NaOCl pH 8 for 500 h (as equivalent to the dose of 1,000,000 ppm·h). It has been reported that NaOCl at pH 8 had stronger oxidation capability than that at pH 10, which has a greater influence on the physiochemical properties of membrane [14,19]. Similar to the A membrane, the number of pores on the B and C membrane surfaces also gradually increase with the prolonged NaOCl aging time. 

The cross-section morphologies of these membranes were shown in Figure S2. The A membrane consisted of a thin layer of finger-like structure and a thick layer of sponge-like structure while B membrane had a homogenous sponge-like structure. The reinforced C membrane consisted of a finger-like structure on top of a braided tubing supporting layer. Despite of the different structure, the cross-section morphologies of these membranes presented no obvious changes under any NaOCl exposure conditions. Therefore, NaOCl aging has a significant impact on membrane surface morphology, which might affect the surface functionality and hydrophilicity as discussed below.

#### 3.1.2. Chemical Compositions of the NaOCl-Aged Membrane Surfaces

**Figure 2:**
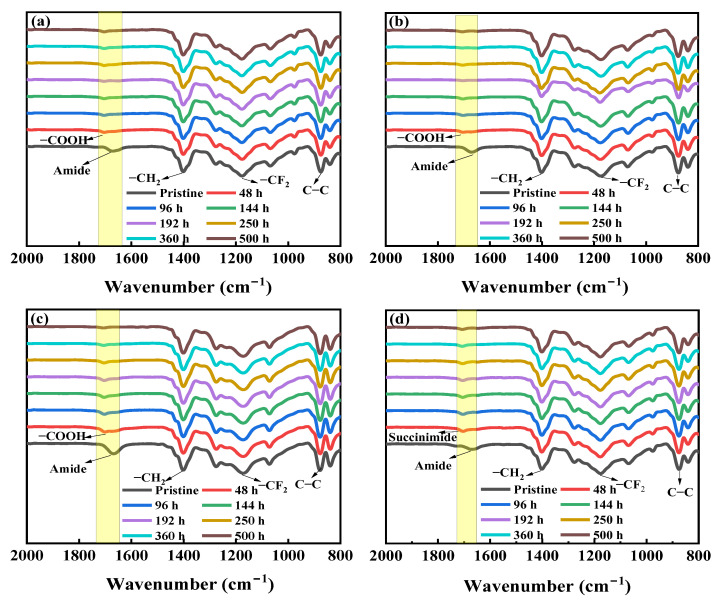
FTIR spectra of the pristine and NaOCl₋exposed membranes. (**a**) A membrane, NaOCl pH = 10; (**b**) B membrane, NaOCl pH = 10; (**c**) C membrane, NaOCl pH = 10; and (**d**) A membrane, NaOCl pH = 8.

The changes in surface functional groups of the NaOCl-aged membranes were determined using ATR-FTIR spectrometer, as shown in Figure 2. Three PVDF pristine membranes presented similar absorption peaks. Moreover, the functional groups of these membranes after NaOCl exposure also showed similar variation trend. Therefore, the A membrane was taken as an example to illustrate the variations of functional groups during aging process. For the pristine membrane, three characteristic absorption peaks at 877 cm^−1^, 1181 cm^−1^, and 1401 cm^−1^ are assigned to the skeletal vibration of C-C bond, stretching vibration of -CF_2_, and stretching vibration of -CH_2_, respectively, which are typical absorption peaks of the PVDF membrane [28]. The absorption peak at 1671 cm^−1^ is attributed to C=O stretching vibration of amide group, indicating the existence of hydrophilic additive polyvinylpyrrolidone (PVP) [13]. Interestingly, the peak at 1671 cm^−1^ disappeared and a new peak at 1703 cm^−1^ formed after a short exposure time of 48 h (as equivalent to the dose of 96,000 ppm·h). This can be attributed to the degradation of PVP by NaOCl in alkaline condition, resulting in pyrrolidone ring opening and formation of carboxylic acid groups [13,14,17]. When the exposure time increased from 48 h to 500 h, the intensity of the peak at 1703 cm^−1^ gradually decreased (this phenomenon is more obvious in C membrane case), possibly ascribing to the further degradation and releasing of PVP. Gao et al. [12] reported that during PVDF membrane aging process, PVP was first degraded thoroughly followed by the decrease in C-F peak intensity due to defluorination and oxygenation reaction at NaOCl pH 12 condition. However, there was no obvious variations in the characteristic peaks of PVDF with the prolonged exposure time in the present study, suggesting that the C-F bond was stable in the NaOCl solution at pH 10. In the case of NaOCl pH 8 condition, the FTIR spectra of the NaOCl-aged membranes presented the similar characteristic peaks as those after exposure to NaOCl at pH 10 (Figure 2d). The appearance of the peak at 1703 cm^−1^ represents the formation of succinimide groups due to the oxidation of PVP by radicals at acid and neutral conditions [13,19]. A similar phenomenon has been reported in a previous study, where a new shoulder peak at 1700 cm^−1^ was identified to represent succinimide group during the treatment of PES/PVP membrane in NaOCl solution at pH 6 and 8 [12].

#### 3.1.3. Changes in Surface Hydrophilicity

**Figure 3:**
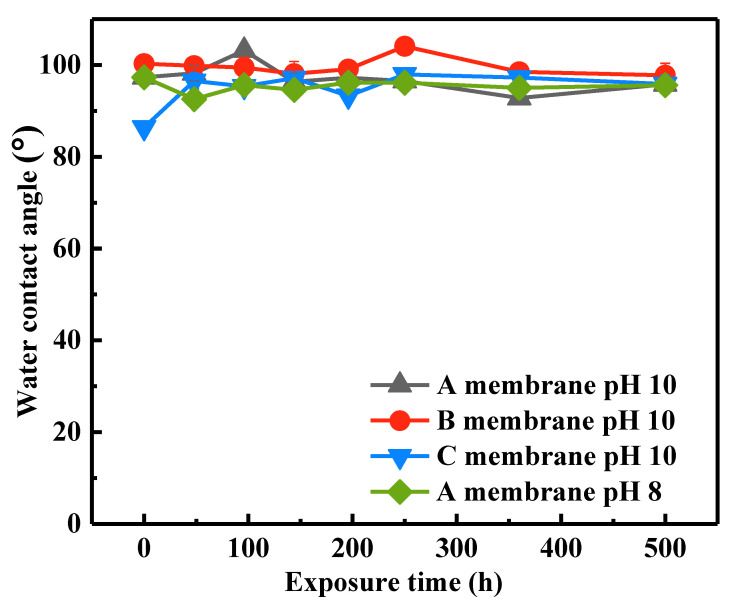
Water contact angles of the pristine and NaOCl₋exposed Membranes.

The changes in surface hydrophilicity of the membranes after NaOCl exposure were determined by dynamic water contact angles (WCAs), as presented in Figure 3. The WCAs of pristine A, B, and C membranes were 97.3°, 100.2° and 86.4°, respectively. The pristine C membrane was more hydrophilic in comparison with the A and B membranes, which might because it has higher concentration of hydrophilic additive. This was evidenced by higher peak intensity of amide group observed in FTIR spectra. After exposure to NaOCl for various time, the WCAs of these membranes only changed slightly. The WCAs of A and B membranes decreased from 97.3° and 100.3° to 95.8° and 97.8° after exposure to NaOCl for 500 h, respectively, whereas the WCA of the C membrane increased from 86.4° to 96.5° after exposure to NaOCl for 48 h and then it maintained consistent at around 96° during subsequent different NaOCl aging periods. As discussed in FTIR section, NaOCl treatment at alkaline condition can degrade PVP and result in the ring opening of pyrrolidone and formation of carboxylic acid groups. On one hand, the degradation and release of PVP from the membrane surface might lead to an increase in membrane hydrophobicity. On the other hand, the formation of carboxylic acid groups can improve membrane hydrophilicity. Thus, under the combination of these two effects, the WCAs of the A and B membranes presented slight fluctuations during different NaOCl aging periods. The increase in the WCA of the C membrane was mainly due to the degradation and dissolution of more hydrophilic additive PVP. For the NaOCl pH 8 case, the WCAs of the A membrane showed few changes after exposure to NaOCl solution for different periods, which was similar with NaOCl pH 10 case. To summarize, NaOCl degraded the hydrophilic additive of membranes, which influenced membrane surface hydrophilicity. The changes in surface functional groups and hydrophilicity might impact membrane separation performance and fouling behavior, which will be discussed in Section 3.2. 

#### 3.1.4. Effects of NaOCl Exposure on Membrane Mechanical Properties

**Figure 4:**
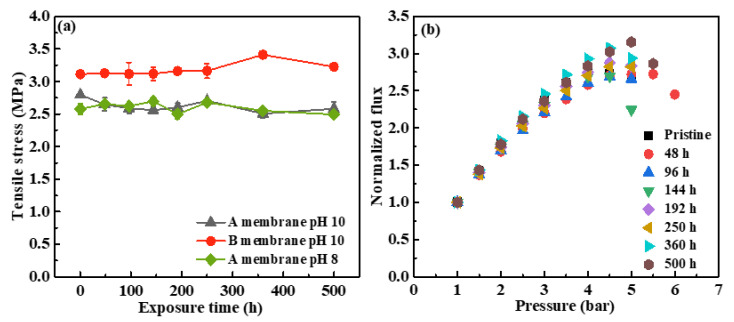
Changes in membrane mechanical properties with NaOCl exposure time. (**a**) Changes in the tensile stress of A and B membranes with NaOCl exposure time; and (**b**) changes in the collapse pressure of the C membrane with NaOCl exposure time.

Figure 4 shows the changes in mechanical properties of these membranes with NaOCl exposure time. The tensile stresses of pristine A and B membranes were 2.79 ± 0.03 Mpa and 3.11 ± 0.05 Mpa, respectively. After exposure to NaOCl at pH 10, the tensile stresses of A and B membranes presented very minor changes. The tensile stress of the A membrane slightly decreased to 2.58 ± 0.09 Mpa while that of B membrane slightly increased to 3.22 ± 0.04 Mpa after exposure to NaOCl for 500 h. In addition, there was no significant difference between the tensile stresses of A membranes after treatment by NaOCl at pH 8 and pH 10, which verified the outstanding mechanical property of the membranes. As shown in Figure 4b, the normalized flux of C membranes increased linearly with the pressure increasing. The collapse pressure of the pristine C membrane was determined at around 4.5 bar. The NaOCl aging process did not change the collapse pressure of C membrane, indicating the robust mechanical property of C membrane. From above discussion, these PVDF hollow fiber membranes had robust mechanical strength, and NaOCl aging treatment did not change their mechanical properties. 

### 3.2. Separation Performance and Fouling Behavior of NaOCl-Exposed Membranes

#### 3.2.1. Pure Water Permeability and HA Rejection of the Pristine and NaOCl-Exposed Membranes 

**Figure 5:**
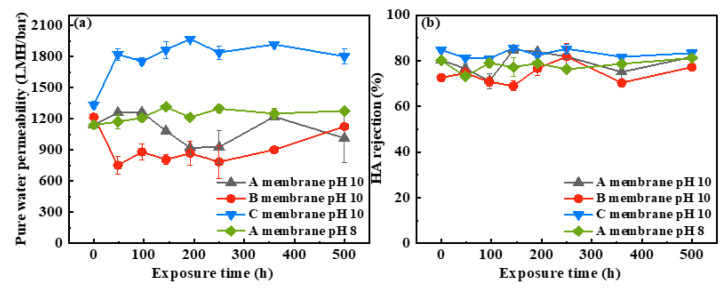
(**a**) Pure water permeability and (**b**) HA rejection of A, B, and C membranes as a function of NaOCl exposure time.

As shown in Figure 5a, the PWP of the A membrane showed a fluctuation with the prolonged exposure time and slightly decreased from 1141 LMH/bar to 1013 LMH/bar after exposure to NaOCl pH 10 for 500 h. For the B membrane, the PWP first decreased from 1216 LMH/bar to 752 LMH/bar after 48 h exposure and then gradually increased to 1125 LMH/bar after 500 h exposure. The PWP fluctuation of the A and B membranes during NaOCl pH 10 treatment can be ascribing to the comprehensive impacts of the following two aspects. On one hand, more pores were observed with NaOCl aging progress (Figure 1), which could increase the PWP. On the other hand, the gradually degradation and dissolution of PVP as well as formation of hydrophilic carboxylic acid groups changed the surface functionality and hydrophilicity, which would affect membrane permeance. For the NaOCl pH 8 case, the PWP of the A membrane gradually increased from 1141 LMH/bar to 1276 LMH/bar after exposure to NaOCl solution for 500 h. This may be due to the stronger oxidation ability of NaOCl at pH 8, leading to increase in pores and enlargement of pore size. Compared with the A and B membranes, the pristine C membrane with thinner separation layer and higher hydrophilicity presented a higher PWP [29,30]. In contrast with the fluctuated PWP of the A and B membranes, the PWP of the C membrane first increased obviously from 1334 LMH/bar to 1822 LMH/bar after 48 h exposure and then it maintained at 1800~1900 LMH/bar during subsequent NaOCl treatments. As observed in Figure 3, the hydrophilicity of the C membrane reduced after 48 h exposure to NaOCl, which might have a negative impact on the water flux. Thus, the increase in PWP of the C membrane after NaOCl treatment was mainly ascribed to the increased number of pores and enlargement of pore size. At longer exposure time, the slight changes in the PWP of the C membrane were mainly due to the insignificant changes in pores and surface hydrophilicity. 

Retention ability is another essential factor for assessing the separation performance of membrane. HA, existing widely in natural water and wastewater, was used as a model organic matter to evaluate the retention ability of the pristine and NaOCl-exposed membranes. As presented in the Figure 5b, the HA rejections of the pristine the A, B, and C membranes were 80%, 72%, and 84%, respectively. The pristine B membrane showed the lowest HA rejection ascribing to its largest pore size (Figure 1). Under various NaOCl exposure conditions (including different time and pH), the HA rejections of these three NaOCl-aged membranes presented slight fluctuations and were similar to those of pristine membranes. Although the number of pores and pore size increased with the prolonged NaOCl exposure time, the insignificant changes in HA rejections of these NaOCl-aged membranes mainly ascribed to the accumulation of the HA layer on the membrane surface, which formed a new selective layer. Our results suggest that these three commercial PVDF hollow fiber membranes have robust chemical stability in NaOCl solution. The separation performance of these membranes showed little change after NaOCl exposure except for 1.4-fold increase in the PWP of C membrane was observed. The results are different with previous studies about PES membranes [14,19], where the normalized flux of the NaOCl-aged PES membranes at pH 7~8 increased by ~6 times. This confirms that PVDF membrane is more tolerant to NaOCl aging in comparison with PES membrane. 

#### 3.2.2. Fouling Behaviors of the Pristine and NaOCl-Exposed Membranes

**Figure 6:**
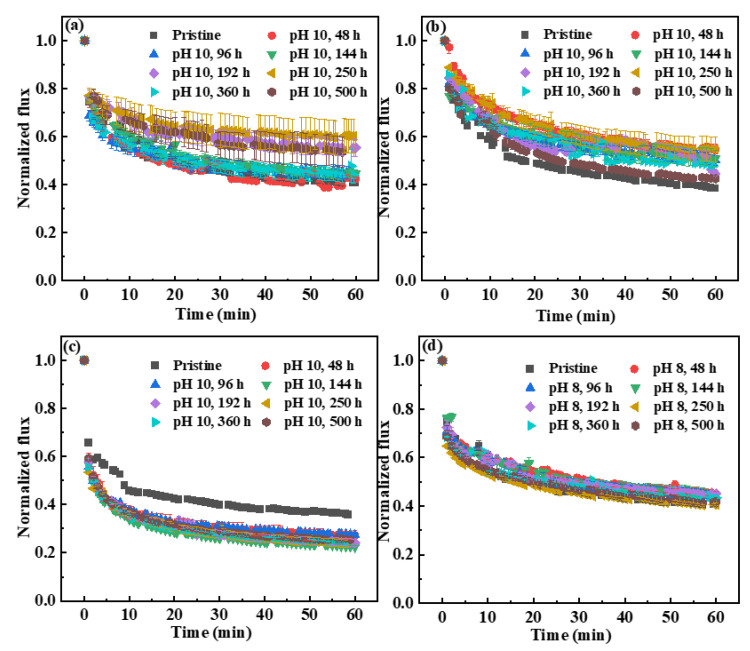
The fouling behaviors of the pristine and NaOCl-exposed membranes. (**a**) A membrane, NaOCl pH 10; (**b**) B membrane, NaOCl pH 10; (**c**) C membrane, NaOCl pH 10; and (**d**) A membrane, NaOCl pH 8.

The effects of NaOCl exposure on membrane fouling behaviors were evaluated using HA as a model foulant on a bench-scale crossflow setup. As shown in Figure 6, the normalized flux of the pristine and NaOCl-exposed membranes showed a sharp decline at the beginning of the test and then it gradually decreased with the time increasing until achieved a pseudo-stable stage. However, the decline extent of the normalized flux was different. It is interesting to note that the antifouling properties of A and B membranes were slightly improved after exposure to NaOCl pH 10, which would benefit the practical applications. The normalized flux of A and B membranes decreased to 0.41~0.60 and 0.38~0.55 after exposure to NaOCl pH 10 for various time, respectively, whereas the normalized flux of pristine membranes decreased to 0.41 and 0.38, respectively (Figure 6a,b). However, the antifouling performance of NaOCl-exposed membranes under pH 8 condition was not as good as that under pH 10 condition. The normalized flux of A membranes after exposure to NaOCl pH 8 showed similar variation decline trend with the pristine membrane (Figure 6d). This might be due to the formation of different functional groups on membrane surface under different NaOCl pH conditions. In our study, the observation of slight improvement of the antifouling properties of the NaOCl-aged A and B membranes was contrary to the previous reports. Zhou et al. reported that NaOCl pH 8-aged PES/PVP membranes presented severe membrane fouling with the HA filtration, while almost no change in permeate flux was observed at pH 10 condition except for a serious flux decline after 30 day-exposure [19]. They ascribed the severe membrane fouling behavior of the NaOCl-aged PES/PVP membranes to membrane pore blocking by hydrophobic HA molecules, which was caused by fast leaching rate of PVP, enlargement of membrane pores, and a fast increase in water permeability. In our study, although membrane pores increased after NaOCl exposure, the hydrophilicity and PWP of the A and B membranes only changed slightly. The improvement of antifouling performance of NaOCl-aged A and B membranes might be due to the formation of carboxylic acid groups (as indicated in FTIR spectra). 

In contrast, the NaOCl-aged C membranes presented faster flux decline in comparison with the pristine membrane. The normalized flux decreased to 0.24~0.36 after exposure to NaOCl for various times (Figure 6c). As discussed in Section 3.1.3, the WCA of C membrane increased obviously after exposure to NaOCl for a short period of 48 h (Figure 3). The increase in membrane hydrophobicity resulted in the easier deposition of HA on the membrane surface via hydrophobic-hydrophobic interaction. Moreover, the PWP of C membrane was increased 1.4 times after exposure to NaOCl for 48 h. The increase in water permeability could allow more HA molecules to enter into membrane pores, leading to pore clogging. Thus, the normalized flux of the NaOCl 48 h-aged C membrane decreased faster than that of the pristine membrane. With the exposure time prolonged (48~500 h), the permeate flux of the C membrane exhibited similar decline extent with the NaOCl 48 h-aged one, which mainly resulted from minor changes in hydrophilicity and water permeability during long-term exposure.

**Figure 7:**
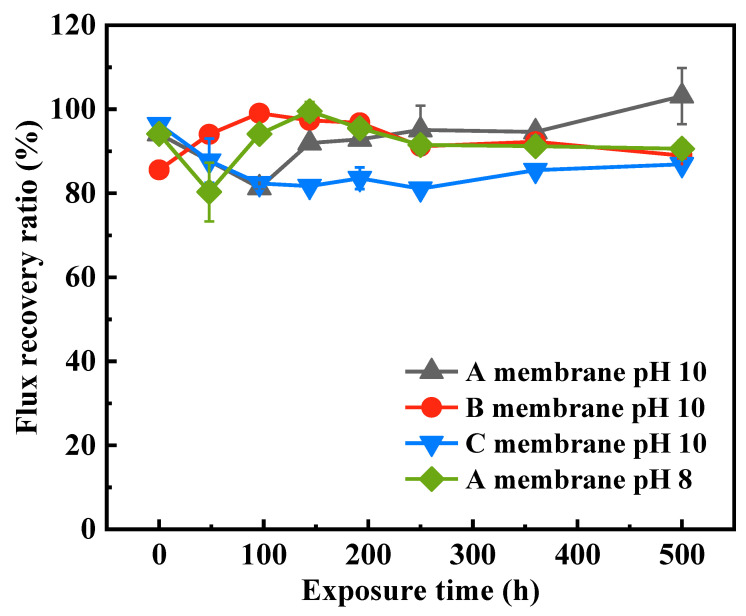
Flux recovery ratios of NaOCl-aged membranes as a function of exposure time.

To further evaluate whether the fouling of the pristine and NaOCl-exposed membranes is reversible or not, physical cleaning was carried out, and the flux recovery ratios (FRRs) of these membranes were shown in Figure 7. The FRRs of all these NaOCl-exposed membranes were higher than 80%, indicating that the deposition of HA on membrane surface was mainly reversible. For the A and B membranes exposed to NaOCl solution at various times, the FRRs presented fluctuation. The FRR of A membrane after exposure to NaOCl pH 10 for 500 h was 103%, which was higher than that of the pristine A membrane (94%). The B membrane after NaOCl pH 10 treatment for 500 h and the A membrane after NaOCl pH 8 treatment for 500 h showed similar FRRs with those of pristine membranes. By contrast, the FRRs of the NaOCl-aged C membrane were obvious lower than that of the pristine membrane, which was consistent with the normalized flux variation trend. This indicated that the NaOCl-aged C membrane induced a relatively higher ratio of irreversible fouling in comparison with the NaOCl-aged A and B membranes during HA filtration. 

In summary, the antifouling performances of A and B membranes were slightly improved after exposure to NaOCl in the alkaline environment, which are beneficial for real applications. The NaOCl treatment on the C membrane, by contrast, aggravated its membrane fouling. In general, all these three commercial PVDF hollow fiber membranes showed good chlorine resistance, and their pure water permeability after HA fouling can be recovered more than 80%.

## 4. Conclusions

In this study, the impacts of NaOCl exposure dose and solution pH on physicochemical properties, filtration performances and fouling behaviors of three widely used commercial PVDF hollow fiber UF membranes were investigated. The main conclusions of this study can be summarized as follows:

(1) The FESEM and ATR-FTIR results demonstrated the degradation and leaching out of hydrophilic additive PVP from the membranes after exposure to NaOCl. The number of pores on the membrane surfaces increased with a prolonged exposure time. The ATR-FTIR results indicated that the degradation and dissolution of PVP by NaOCl in alkaline condition led to the formation of carboxylic acid group through ring opening of pyrrolidone, while a succinimide group was formed at pH 8 condition.
(2) The WCAs of the SMM-1010 and MEMCOR^®^ CS II membranes showed insignificant change with the various exposure time, whereas the WCA of the ZeeWeed 500 membrane increased after exposure to NaOCl. These three membranes presented robust mechanical properties during NaOCl aging process, which is independent of exposure dose and solution pH, ascribing to the stable C-F bonds.
(3) The PWP of the SMM-1010 and MEMCOR^®^ CS II membranes slightly declined after exposure to NaOCl pH 10 for 500 h, while that of the NaOCl aged-ZeeWeed 500 membrane increased by ~1.4-fold. The HA rejections of these membranes had insignificant changes during NaOCl aging process. The antifouling performances of SMM-1010 and MEMCOR^®^ CS II membranes were slightly improved after exposure to NaOCl in the alkaline condition, whereas NaOCl aging aggravated ZeeWeed 500 membrane fouling.

Our findings suggest that chemical cleaning with NaOCl solution is better performed under alkaline conditions (pH 10). All of these three commercial PVDF hollow fiber UF membranes present good chlorine resistance. The correlation between the membrane physicochemical properties and performance should be further understood to optimize the cleaning condition in the future.

## Figures and Tables

**Table 1:** The Characteristics of the commercial hollow fiber UF membranes.

No	Manufacturer	Model number	Structure	Outer Diameter (mm) ^a^	Inner Diameter (mm) ^a^	Mean Pore Size (μm) ^b^
A	Memstar, Singapore	SMM-1010	Self-supported membrane	1.22 ± 0.02	0.63 ± 0.03	<0.1
B	DuPont, Wilmington, USA	MEMCOR^®^ CS II (S10N)	Self-supported membrane	1.08 ± 0.02	0.50 ± 0.02	0.04
C	Suez, Paris, France	ZeeWeed 500	Reinforced membrane	1.98 ± 0.01	0.95 ± 0.02	0.04

^a^ The outer and inner diameters of the hollow fibers were measured by an optical microscope (CSW-H4KACL, China). ^b^ The mean pore size data were provided by the manufacturers.

## Data Availability

The data presented in this study are available on request from the corresponding author.

## Supplementary Materials

The following supporting information can be downloaded at: https://www.mdpi.com/article/10.3390/membranes12100965/s1, Figure S1: Schematic illustration of the bench-scale cross-flow filtration system; Figure S2: FESEM images of the cross-section morphologies of pristine and NaOCl-exposed membranes. (a) A membrane, NaOCl pH=10; (b) B membrane, NaOCl pH=10; (c) C membrane, NaOCl pH=10; and (d) A membrane, NaOCl pH=8; 1, 2, 3, and 4 represent exposure time 0, 48, 192, and 500 h, respectively.
