# Supplementary material for: Unveiling the Impacts of Sodium Hypochlorite on the Characteristics and Fouling Behaviors of Different Commercial Polyvinylidene Fluoride Hollow Fiber Membranes
Source: Membranes (Basel). 2022 Oct 1;12(10):965. doi: 10.3390/membranes12100965 (PMC9607576; doi:10.3390/membranes12100965)
Supplement: Supplementary file 1 [file membranes-12-00965-s001.zip › membranes-1945405-supplementary.pdf]

Supporting Information for

Unveiling the Impacts of Sodium Hypochlorite on the Characteristics and Fouling Behaviors of Different Commercial Polyvinylidene Fluoride Hollow Fiber Membranes

Muqiao Han [1,2], Qi Han [3], Shanwei Wu [3], Hu Xiao [3], Lei Zhang [3], Yibo Lin [1,2], Fangang Meng [1,2] and Shanshan Zhao [1,2,*]

[1] School of Environmental Science and Engineering, Sun Yat-sen University, Guangzhou 510006, China
[2] Guangdong Provincial Key Laboratory of Environmental Pollution Control and Remediation Technology, Guangzhou 510006, China
[3] Guangzhou Jinrongtao Environmental Protection Technology Co., Ltd., Guangzhou, 511400, China
* Correspondence: zhaoshsh7@mail.sysu.edu.cn

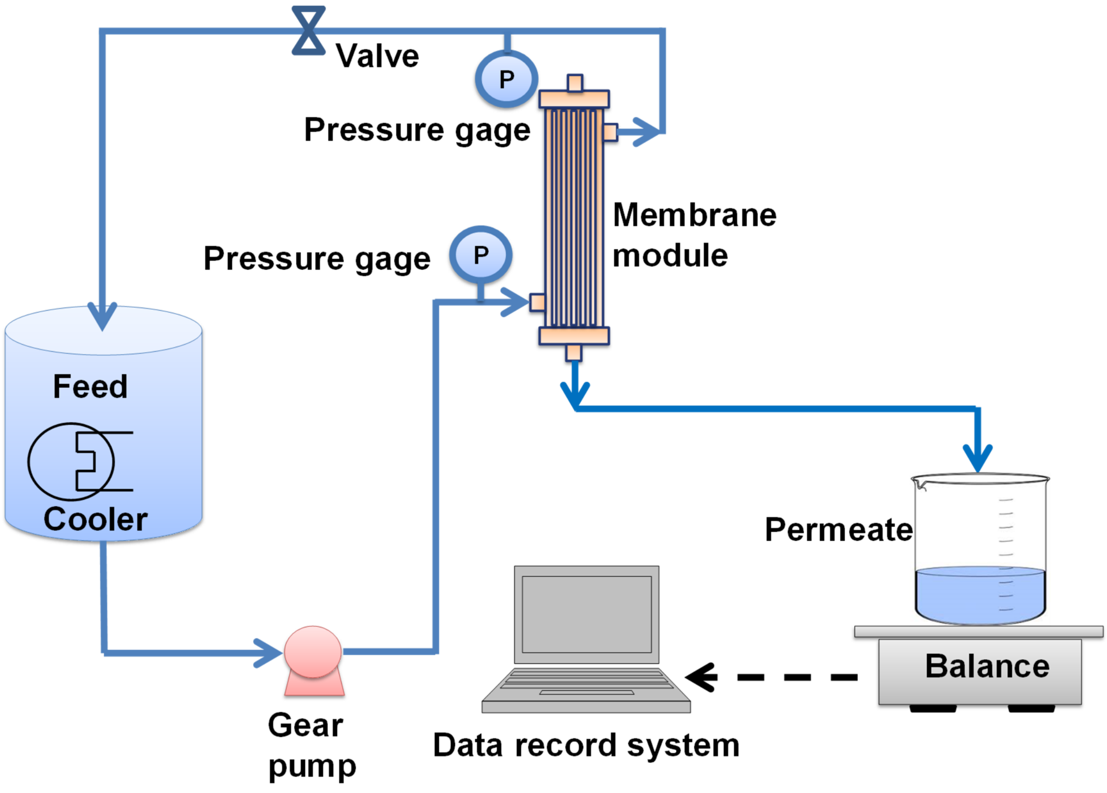

Figure S1. Schematic illustration of the bench-scale cross-flow filtration system.

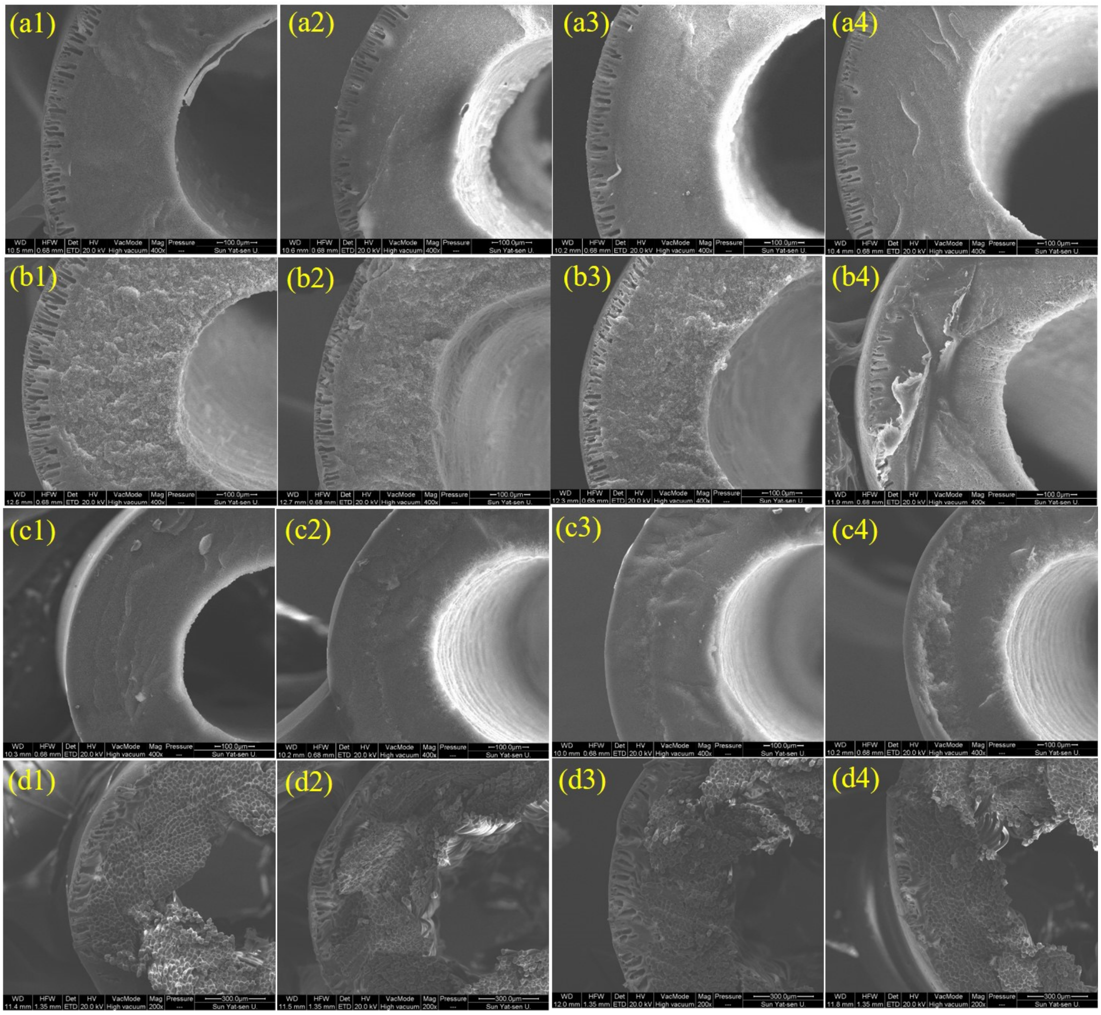

Figure S2. FESEM images of the cross-section morphologies of pristine and NaOCl-exposed membranes. (a) A membrane, NaOCl pH=10; (b) B membrane, NaOCl pH=10; (c) C membrane, NaOCl pH=10; and (d) A membrane, NaOCl pH=8; 1, 2, 3, and 4 represent exposure time 0, 48, 192, and 500 h, respectively.
